# A prospective longitudinal study of risk factors for abdominal aortic aneurysm

**DOI:** 10.14814/phy2.16130

**Published:** 2024-06-30

**Authors:** Benjamin S. Stacey, Jun Seok Cho, Damien Lanéelle, Mohammad Bashir, Ian M. Williams, Michael H. Lewis, Damian M. Bailey

**Affiliations:** ^1^ Neurovascular Research Laboratory, Faculty of Life Sciences and Education University of South Wales Pontypridd UK; ^2^ Department of Surgery Royal Free Hospital London UK; ^3^ UNICAEN, CHU Caen Normandie, Vascular Medicine Unit, INSERM, COMETE Caen France; ^4^ Department of Surgery University Hospital Wales Cardiff UK

**Keywords:** abdominal aortic aneurysm, pathogenesis, risk factors

## Abstract

The aim of this study was to identify risk factors for abdominal aortic aneurysm (AAA) from the largest Welsh screening cohort to date. Patients were recruited from 1993 (to 2015) as part of the South East Wales AAA screening programme through general practitioners. Demographic data and risk factors were collected by means of a self‐report questionnaire. Statistical tests were performed to determine whether associations could be observed between AAA and potential risk factors. Odds ratios (OR) were also calculated for each of the risk factors identified. A total of 6879 patients were included in the study. Two hundred and seventy‐five patients (4.0%) presented with AAA, of which 16% were female and 84% were male. Patients with AAA were older than the (no AAA) control group (*p* < 0.0001). The following risk factors were identified for AAA: family history of AAA (*p* < 0.0001); history of vascular surgery (*p* < 0.0001), cerebrovascular accident (*p* < 0.0001), coronary heart disease (*p* < 0.0001), diabetes (*p* < 0.0001), medication (*p* = 0.0018), claudication (*p* < 0.0001), smoking history (*p* = 0.0001) and chronic obstructive pulmonary disorder (*p* = 0.0007). AAA is associated with classical vascular risk factors, in addition to other less‐well‐documented risk factors including previous vascular surgery. These findings have practical implications with the potential to improve future clinical screening of patients in order to reduce AAA mortality.

## 1 INTRODUCTION

An abdominal aortic aneurysm (AAA) is commonly defined as a maximum infrarenal abdominal aortic diameter of ≥3.0 cm, representing more than two standard deviations above the mean diameter of 1.79–1.93 cm for men (Rogers et al., 2013), as measured by ultrasonography (US) or computed tomography angiography (CTA) (Schanzer & Oderich, 2021). While most often asymptomatic, AAA will slowly progress to rupture with a high mortality rate of 5% of patients per year among those with an AAA diameter of >5.5 cm (Parkinson et al., 2015) or 100% fatal in non‐treated cases (Hultgren et al., 2016). Given that the risk of rupture for patients under surveillance with small (3.0–4.4 cm) or medium‐sized (4.5–5.4 cm) AAAs is low, estimated in the order of <0.5% per year (Oliver‐Williams et al., 2019), early detection by US/CTA screening for AAA provides the greatest ability to potentially prevent AAA‐related death by allowing for early intervention with open surgery or endovascular repair when the aneurysm reaches a diameter of ≥5.5 cm (Kent, 2014). Second to this, identifying risk factors and their relative weight in the genesis and progression of AAA pathology remains of the upmost importance.

As an arterial disease, the pathophysiology of AAA shares many of the same risks factors of atherosclerotic occlusive pathology, namely, age, smoking, and family history (Kent et al., 2010). Accordingly, the risk of AAA increases with age, where the incidence per 100,000 people per year was 55, 85, and 298 in those aged 65–74 years, 75–85 years, and ≥85 years, respectively (Howard et al., 2015). Additionally, this increase in AAA risk is observed more in men, where the delay in AAA development in women has been attributed to the protective effects of estrogen on the cardiovascular system prior to menopause (Li et al., 2013). Smoking remains the leading modifiable risk factor for AAA, representing 75% of all AAA cases >4.0 cm in diameter (Lederle et al., 2000) and with reported odds ratios between 2.3 and 13.7 (Forsdahl et al., 2009; Jahangir et al., 2015; Wanhainen et al., 2005), smoking yields a stronger association to AAA than that observed between smoking and chronic obstructive pulmonary disease (COPD) and coronary heart disease (CHD) (Lederle et al., 2003; Pujades‐Rodriguez et al., 2015). Lastly, as with common pathophysiology, those with a first‐degree relative (i.e., parent, offspring, or sibling) with an AAA are at greater risk of developing an AAA in their lifetime with an observed increase in growth rate and higher incidence of rupture, despite no apparent difference in aneurysm morphology (Larsson et al., 2009; van de Luijtgaarden et al., 2014, 2017).

In England and Wales, a national screening programme for AAA was introduced in 2009 and 2013, respectively, with regional screening programmes in the UK established much earlier: Chichester in 1984 (Scott et al., 1995), Gloucester in 1990 (O'Kelly & Heather, 1989), Huntington in 1991 (Wilmink, Quick, Hubbard, & Day, 1999), and South Wales in 1993. The present work examines data obtained from the AAA screening between 1993 and 2015 in South Wales by comparing demographics (age and sex) and risk factors within control subjects (negative screening) and AAA patients (positive screening). Secondly, we also sought to quantify the relative frequency of risk factors according to aneurysm size.

## 2 METHODS

### 2.1 Ethical approval

Ethical approval was granted by the National Research Ethics Service committee London—South East (Reference: 14/LO/1958, IRAS project ID: 141235). All procedures were carried out in accordance with the Declaration of Helsinki of the World Medical Association with written informed consent obtained from all participants (Williams, 2008).

### 2.2 Design

This is a retrospective cohort study based on data collected prospectively between 1993 and 2015 from an AAA screening programme in East Glamorgan General Hospital in South East Wales.

### 2.3 Screening programme

The screening team contacted general practitioners (GP) in the region to obtain a list of eligible members of the population. The eligibility criteria evolved over time to respond to changing knowledge: from 1993 to 2003, men aged 60–80 and women aged 65–80; from 2003 to 2005, men and women aged 65–80; and from 2005 to 2015, only men aged 65. Participants were invited to the hospital for screening which included measurement of AAA diameter via US and completion of a health‐history questionnaire. Patients with AAA were subdivided into small (3.0–4.4 cm), medium (4.5–5.4 cm), and large (≥5.5 cm) AAA according to national AAA screening criteria (NHS, 2013).

### 2.4 Questionnaire

The health‐history questionnaire included patient demographics (age and sex), medical history (hypertension, coronary artery disease, cerebrovascular incidents, chronic obstructive pulmonary disease, diabetes mellitus, and claudication), vascular surgical history (surgery of arteries or veins), medication history, smoking history, and family history (first‐degree relatives with AAA).

### 2.5 Statistical analysis

Data were analyzed using the Statistics Package for Social Scientists (IBM SPSS Statistics Version 29.0). Distribution normality was confirmed using repeated Shapiro–Wilk W tests. Significance for all two‐tailed tests was established at *p* < 0.05 and data presented as mean ± SD unless otherwise indicated. Chi‐squared test or Fisher's exact test were used for categorical data relative frequencies and two‐tailed unpaired *t*‐tests was used for parametric continuous data. Tests were performed to determine whether associations could be observed between AAA and potential risk factors. Odds ratio were also calculated for each of the risk factors identified. Where statistically significant effects (*p* < 0.05) were observed, standardized residuals were used to determine which subgroups were under‐ or over‐represented in terms of the proportions of members with each risk factor.

## 3 RESULTS

### 3.1 Participant uptake

**FIGURE 1:**
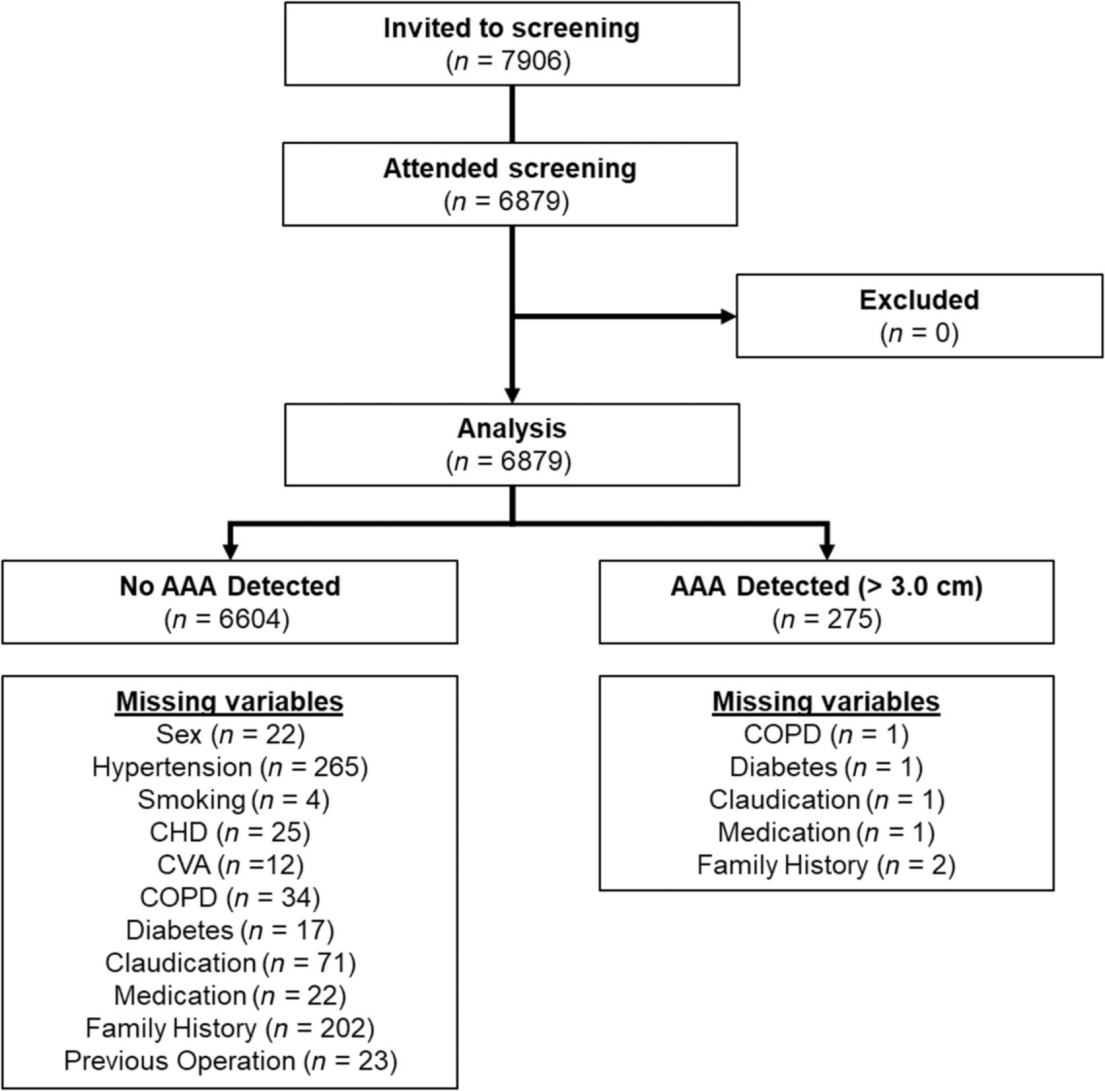
Flow diagram of the process through the phases of the study. Missing variables were due to unanswered questions within the questionnaire. AAA, abdominal aortic aneurysm; CHD, coronary heart disease; CVA, cerebrovascular accident; COPD, chronic obstructive pulmonary disorder; *n*, sample size; SD, standard deviation. *different versus controls (*p* < 0.05).

Of the 7906 participants who received a formal invitation letter to participate in AAA screening between 1993 and 2015, a total of 6879 attended screening and were included into this study (Figure 1).

### 3.2 Demographics

Of the 6879 participants who attended screening, 6604 (96.0%) had no AAA (controls) and 275 (4.0%) participants were identified with AAA (abdominal aortic diameter ≥3 cm). Of these, 166 (60%), 93 (34%), and 16 (6%) were categorized as having a small (3.0–4.4 cm), medium (4.5–5.4 cm), or large (>5.5 cm) AAA. The mean (± standard deviation) age of patients identified with AAA was higher than that of the control group without AAA (72.6 ± 7.4 years vs. 68.7 ± 5.5 years, *p* = <0.001) attributed to the older participants in the small and medium AAA group (*p* = <0.001) but not for the large AAA group (Table 1). There was a higher prevalence of AAA in men compared to women in the combined AAA group (83.6% male and 16.4% female, *p* = <0.001); (Table 1).

**TABLE 1:** Participant characteristics.

Group:	Controls	AAA			
	(*n* = 6604)	Small 3.0–4.4 cm (*n* = 166)	Medium 4.5–5.4 cm (*n* = 93)	Large <5.5 cm (*n* = 16)	All >3.0 cm (*n* = 275)
Demographics					
Age (years ± SD)	68.7 ± 5.5	71.6 ± 6.9*	74.6 ± 7.9*	71.3 ± 8.0	72.6 ± 7.4*
Males *n* (%)	3581 (54.2)	138 (83.1)*	78 (83.9)*	14 (87.5)*	230 (83.6)*
Females *n* (%)	3001 (45.4)	28 (16.9)*	15 (16.1)*	2 (12.5)*	45 (16.4)*
Risk factors					
Hypertensive, *n* (%)	4239 (67)	107 (64)	66 (71)	12 (75)	185 (67)
Smoking history, *n* (%)	1255 (19)	49 (30)*	25 (27)	4 (25)	78 (28)*
CHD, *n* (%)	1478 (22)	60 (36)*	38 (41)*	7 (44)*	105 (38)*
CVA, *n* (%)	527 (8)	26 (16)*	17 (18)*	2 (13)	45 (16)*
COPD, *n* (%)	1526 (23)	46 (28)	38 (41)*	4 (25)	88 (32)*
Diabetes, *n* (%)	657 (10)	29 (17)*	18 (20)*	2 (13)	49 (18)*
Claudication, *n* (%)	1482 (23)	57 (34)*	32 (35)*	5 (31)	94 (34)*
Medication, *n* (%)	5568 (85)	148 (89)	89 (97)*	14 (88)	251 (92)*
Family history, *n* (%)	268 (4)	22 (13)*	15 (16)*	1 (6)	38 (14)*
Prior vascular surgery *n* (%)	743 (11)	46 (28)*	31 (33)*	5 (31)*	82 (30)*

Abbreviations: AAA, abdominal aortic aneurysm; CHD, coronary heart disease; CVA, cerebrovascular accident; COPD, chronic obstructive pulmonary disorder; SD, standard deviation.

*Note*: Data expressed as cumulative frequency (n) and percentage (%) of the given sample population.

* Different versus controls (*p* < 0.05).

### 3.3 Risks factors

**FIGURE 2:**
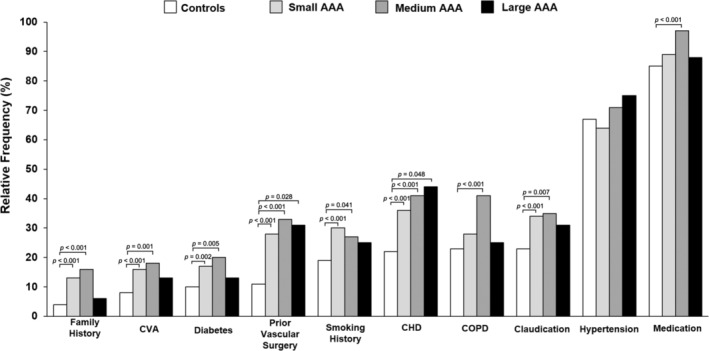
Relative frequency of risk factors between the control group and AAA cohorts. Data expressed as frequency percentages (%) of the given sample population. AAA, abdominal aortic aneurysm; CHD, coronary heart disease; CVA, cerebrovascular accident; COPD, chronic obstructive pulmonary disorder; *different versus controls (*p* < 0.05).

**FIGURE 3:**
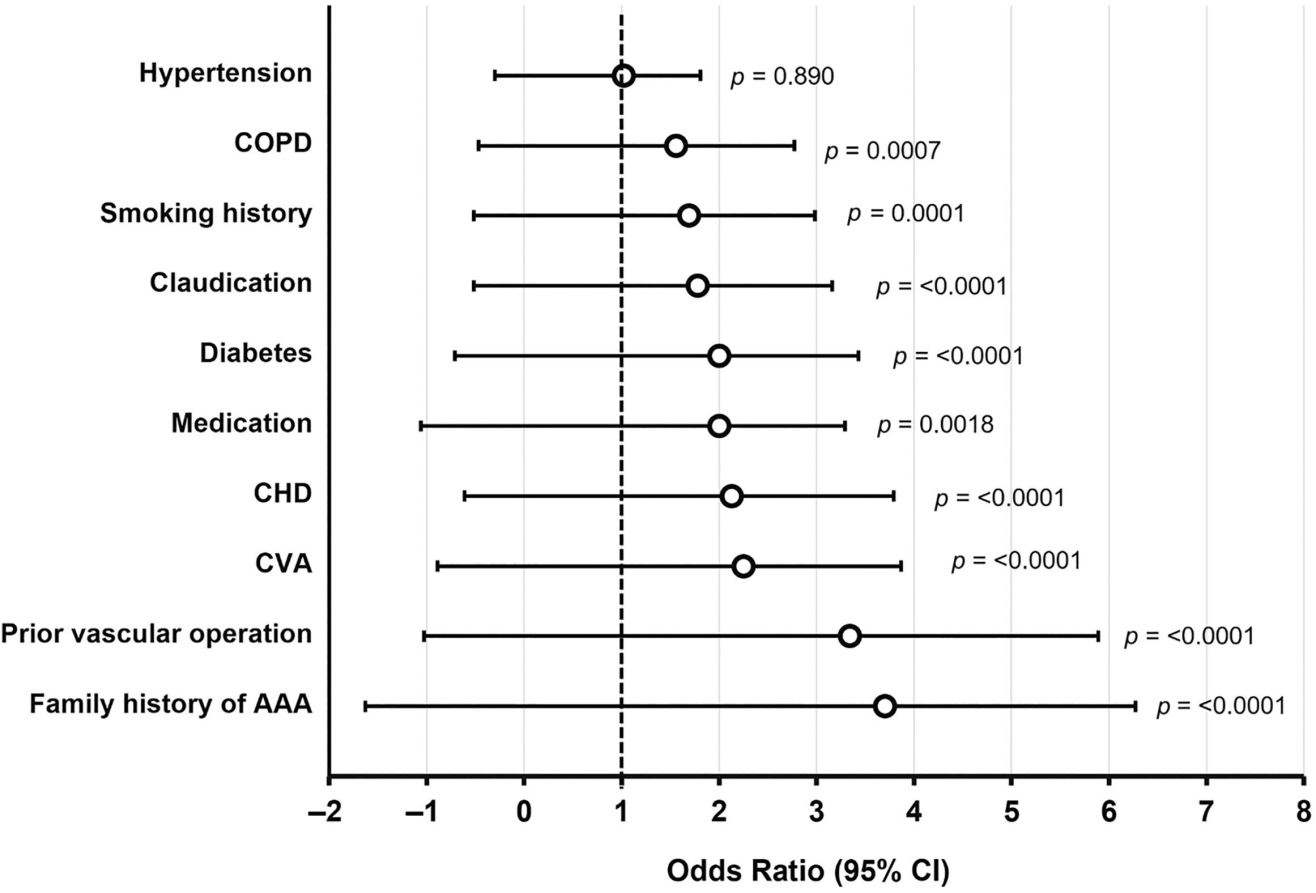
Odd ratios for risk factors associated to abdominal aortic aneurysm (>3.0 cm). Data expressed as odd ratios and 95% confidence intervals. AAA, abdominal aortic aneurysm; CHD, coronary heart disease; CVA, cerebrovascular accident; COPD, chronic obstructive pulmonary disorder.

As expected, classical risk factors were more frequent in the AAA patients than in the control group (*p* = <0.001) with the exception of hypertension (*p* = 0.890) as seen in Table 1 and Figure 2. The risk factor with the highest odd ratio was familial history of AAA (OR 3.70, 95% CI = 2.57–5.33, *p* = <0.0001, Figure 2). Other significant risk factors for AAA included: smoking history (OR 1.69, 95% CI = 1.29–2.21, *p* = 0.0001), coronary heart disease (OR 2.13, 95% CI = 1.66–2.74, *p* < 0.0001), cerebrovascular accident (OR 2.25, 95% CI = 1.62–3.14, *p =* <0.0001), chronic obstructive pulmonary disorder (OR 1.56, 95% CI = 1.21–2.03, *p* = 0.0007), diabetes mellitus (OR 2.00, 95% CI = 1.43–2.71, *p* = <0.0001), claudication (OR 1.78, 95% CI = 1.38–2.30, *p =* <0.0001), surgical history of arteries or veins (OR 3.34, 95% CI = 2.55–4.37, *p* = <0.0001), and regular medication (OR 2.00, 95% CI = 1.29–3.06, *p* = 0.0018) (Figure 3).

## 4 DISCUSSION

The present study is the largest Welsh study to investigate the prevalence and risk factors for AAA to date. With an exceptionally high participant uptake throughout the study period (87%), attributed to screening taking place locally within the community following GP referral, we found the prevalence of AAA to be at 4.0% and associated with classical risk factors including age, male sex, smoking, CHD, CVA, COPD, diabetes mellitus, claudication, medication, prior vascular surgery, and a positive family history of AAA. Collectively, these findings have practical implications with the potential to improve clinical screening of patients in order to reduce AAA mortality.

### 4.1 Age

It is well documented that the risk of developing AAA increases with age (Howard et al., 2015), supported by our findings where the AAA group (all) were older than our control group (~ +4 years). Accordingly, national screening programmes (including that of the present study from 2005 to 2015) have focussed their limited resources in targeting men aged 65 years (NHS, 2011–12; Force et al., 2019). Conversely, given that we observed those with AAA to be much older (72.6 ± 7.4 years) alongside the lower‐than‐expected prevalence of AAA among 65 year‐old‐men (Svensjo et al., 2011), AAA rupture shifting to older age (Choke et al., 2012) and increased life expectancy of the elderly population, future AAA screening strategies may require alteration. Of note, we also observed a higher mean age of the small and medium AAA group compared to the control group (71.6 ± 6.9 and 74.6 ± 7.9 vs. 68.7 ± 5.5 years, respectively) but there was no difference between the large AAA group and control group (71.3 ± 8.0 vs. 68.7 ± 5.5 years, respectively). This may be to inadequate power to detect a difference given the small number of patients in the large AAA group.

### 4.2 Biological sex

The present study observed a higher AAA prevalence in men compared to women (6.0% vs. 1.5%), supporting the evidence that AAA disproportionally affects men (Svensjo et al., 2013) and remains the foremost argument for excluding women from national AAA screening programmes. That women develop AAA at a later age (Sweeting et al., 2012) has been primarily attributed to the protective effects of estrogen on the cardiovascular system prior to menopause (Li et al., 2013). Additionally, male androgens appear to have an adverse effect on AAA formation as seen in AAA rodent models following orchiectomy and administration of dihydrotestosterone to castrated mice (Henriques et al., 2004). Given the smaller aortic diameter in women (Lo et al., 2014) and higher rate of rupture (Vanni et al., 2016), some have called for a smaller threshold for diagnosis and intervention in a bid to increase AAA prognosis among women at a younger age (Ulug et al., 2017). However, this remains controversial given the potential harm of overdiagnosis when using alternative definitions. Indeed, diagnosing AAA using an aortic maximum diameter ≥1.5 times normal infrarenal aortic diameter has shown an AAA prevalence of up to 10% in women (Wanhainen et al., 2001). Furthermore, the higher 30‐day mortality and surgical complications following open or endovascular AAA repair in women (Grootenboer et al., 2010) would argue for a higher threshold for elective surgery.

### 4.3 Familial history of AAA

Since Clifton suggested a familial tendency for AAA development in 1977 (Clifton, 1977), studies have consistently shown family history of AAA to be a strong risk factor for AAA (Lederle et al., 2000). This agrees with our data with the highest OR for AAA of 3.7 and approximately 14% of the patients with AAA had a first‐degree family history of AAA. Of clinical importance, in these individuals, AAA appears earlier and is more likely to rupture, compared to sporadic cases (Sakalihasan et al., 2014) emphasizing the need to screen these patients earlier. While this has been ascribed primarily to epigenetics, namely smoking, diet, and obesity (Bjorck & Wanhainen, 2013), genome‐wide association studies (GWAS) on single nucleotide polymorphisms (SNPs) have identified associations between AAA and: a SNP on chromosome 3p12.3, close to contactin‐3 (CNTN3) gene coding for a cell adhesion molecules; a SNP on chromosome 9q33 located within the *DAB2IP* gene, encoding for an inhibitor of cell growth and survival and a SNP on chromosome 12q13 located within intron 1 of low‐density lipoprotein receptor‐related protein 1 (LRP1) (Elmore et al., 2009; Gretarsdottir et al., 2010). It should be noted that the odd ratios for these studies were low and fail to validate one‐another.

### 4.4 Smoking

Smoking remains one of the largest risk factors for AAA formation (Forsdahl et al., 2009; Jahangir et al., 2015; Wanhainen et al., 2005), as confirmed by our findings (OR = 1.69). Studies have shown that current smokers are more than seven times more likely to have a AAA than age‐matched nonsmokers (Wilmink, Quick, & Day, 1999) and men who smoke more than 25 cigarettes per day have a 15‐fold increased risk of AAA (Wong et al., 2007). The duration of smoking appears to have a larger effect than number of cigarettes smoked per day and importantly, the risk of AAA decreases with smoking cessation (Wilmink, Quick, & Day, 1999). Additionally, those with AAA who continued to smoke had higher growth rates of AAA (MacSweeney et al., 1994). The exact mechanism for this association remains unclear but it is likely associated to the proteolytic degradation of connective tissue, including elastin and collagen, via the tobacco smoke‐induced inhibition of anti‐elastase activity of alpha1‐antitrypsin (Maclay et al., 2012; Segura‐Valdez et al., 2000).

### 4.5 Comorbidities

Our data has shown a higher prevalence of CHD (OR = 2.13), CVA (OR = 2.25), and claudication (OR = 1.78) in patients with AAA compared to patients without AAA. This is in keeping with previous studies which reported high prevalence of co‐existing atherosclerotic vascular disease in AAA patients (Rogers et al., 2013). We also found that previous surgical history on arteries or veins was strongly related to AAA, most likely due to fact that the arterial operations (e.g., coronary arterial bypass grafts) are a consequence of coexisting systemic atherosclerotic vascular disease.

Not limited to the cardiovascular system, we also observed strong associations between AAA and COPD. This is supported by prior research demonstrating a 1.22‐fold increase in AAA risk in patients with COPD, compared to non‐COPD patients (Xiong et al., 2016). Moreover, patients with oxygen dependent COPD present with a 2‐fold increased risk of AAA than those with medical (non‐oxygen dependant) COPD and there appears to a linear dose response to AAA, whereby patients with more severe COPD were more likely to have AAA than patients with less severe disease (19.3% vs. 7.6%) (van Laarhoven et al., 1993).

Surprisingly, our results indicated that diabetes mellitus was a significant risk factor for AAA, whereas other studies have shown a slight protective effect (De Rango et al., 2014). The mechanisms are not fully understood but may involve the action of diabetic therapeutic agents (Nordon et al., 2009). Additionally, while hypertension is a widely considered to be risk factor for atherosclerosis, studies have only demonstrated a weak (odds ratio between 1.24 and 1.46) (Cornuz et al., 2004; Golledge et al., 2006) or in line with our study—a nonexistent relationship (Alcorn et al., 1996; Blanchard et al., 2000) with AAA.

## 5 CONCLUSION

These unique findings have direct practical implications with the potential to improve clinical screening of patients in order to reduce AAA mortality. In addition, the genetic predisposition to AAA may require earlier identification of “at risk” families and targeted screening.

## AUTHOR CONTRIBUTIONS

BSS, JSC, and DMB had full access to all data in the study and take responsibility for the integrity of data and the accuracy of data analysis. JSC, IMW, MHL, and DMB were involved in concept and design. All authors were involved in acquisition, analysis, and interpretation. BSS, JSC, and DMB were involved in drafting of the manuscript. All authors were involved in critical revision of the manuscript for important intellectual content. All authors approved the final version of the manuscript. Agree to be accountable for all aspects of the work in ensuring that questions related to the accuracy or integrity of any part of the work are appropriately investigated and resolved: All authors.

## FUNDING INFORMATION

On behalf of the South East Wales Vascular Network (DMB and IMW), National Cardiovascular Research Network (DMB) and the Société Française de Médecine Vasculaire (DL). DMB is supported by a Royal Society Wolfson Research Fellowship (#WM170007) and Higher Education Funding Council for Wales (Studentship for BSS).

## CONFLICT OF INTEREST STATEMENT

D.M.B. is Editor‐in‐Chief of Experimental Physiology, Chair of the Life Sciences Working Group and member of the Human Spaceflight and Exploration Science Advisory Committee to the European Space Agency, member of the Space Exploration Advisory Committee to the UK Space Agency and Traumatic Brain Injury Committee to the Medical Research Council. D.M.B. is affiliated to the companies FloTBI Inc., BrainEx Inc., and OrgEx Inc. focused on the technological development of novel biomarkers of brain injury in humans.

## Data Availability

Requests for anonymised patient data arising from this research should be directed to Professor Damian Miles Bailey.
